# Functional patterns of healthy human respiratory dynamics by 3D MR spirometry

**DOI:** 10.1007/s00330-025-11838-0

**Published:** 2025-08-08

**Authors:** Nathalie Barrau, Adrien Duwat, Catalin Fetita, Killian Sambourg, Antoine Beurnier, Claire Pellot-Barakat, Angéline Nemeth, Brice Fernandez, Tanguy Boucneau, Vincent Lebon, Xavier Maître

**Affiliations:** 1 Université Paris-Saclay, CEA, CNRS, Inserm, BioMaps, Orsay, France; 2 Institut National des Télécommunications, ARTEMIS, Evry, France; 3 Department of Physiology and Functional Explorations, Hôpital Bicêtre, APHP, Kremlin-Bicêtre, France; 4 GE HealthCare, Buc, France

**Keywords:** Functional lung MRI, Quantitative imaging, Prospective clinical study, Regional ventilation, Gravity lung dependence

## Abstract

**Objectives:**

Functional pulmonary MRI can assess the pathophysiology of regional ventilation, provided that nominal ventilatory patterns are characterised as a baseline. This study investigates common features and their associated gravity dependence using 3D MR spirometry in freely breathing healthy volunteers.

**Materials and methods:**

Repeated dynamic lung MR acquisitions were performed at 3 T on 25 healthy volunteers breathing freely in the supine and prone positions. Three-dimensional maps of tidal volumes (TV), peak expiratory flows (SPEF), expiratory flows at 25% of tidal volume (SEF25), and anisotropy deformation index (ADI) were inferred and normalised. Intra- and inter-volunteer reproducibility was evaluated using percentage differences (PD) and intraclass correlation coefficients (ICC), while gravity dependence was tested using a paired Wilcoxon test.

**Results:**

Twenty-five volunteers (mean age, 45 years ± 17 [standard deviation]; 15 males) were included. Respiratory parametric maps are found spatially inhomogeneous throughout each volunteer’s lung, with large coefficients of variation ranging between 30 and 63%. Yet, the main respiratory patterns are shared among volunteers with common features primarily governed by lung gravity dependence for TV, SPEF, and SEF25 (*p* < 0.05). Spirometry biomarkers are globally repeatable despite intrinsic physiological variabilities (median PD: 5.7–9.2%, ICC: 0.71–0.88), and fairly repeatable locally after normalisations (median PD: 11–19%, ICC: 0.78–0.90).

**Conclusion:**

3D MR spirometry exhibits shared respiratory features between individuals with gravity dependence. Intra-volunteer repeatability and global accuracy were found, demonstrating the reliability of the technique. A new baseline is established for regional lung pathophysiology.

**Key Points:**

***Question*** *Nominal ventilatory patterns in free breathing need to be characterised for regional pathophysiology.*

***Findings*** *Functional ventilatory maps showed significant inhomogeneity, but key patterns, primarily governed by gravity, were consistent across subjects. 3D MR spirometry demonstrated reliability despite physiological variability.*

***Clinical relevance*** *3D MR spirometry is a reliable technique for absolute quantification of the regional ventilation and its dynamic. Nominal spatial patterns of the ventilation should be considered for assessing regional pathophysiology in free breathing.*

**Graphical Abstract:**

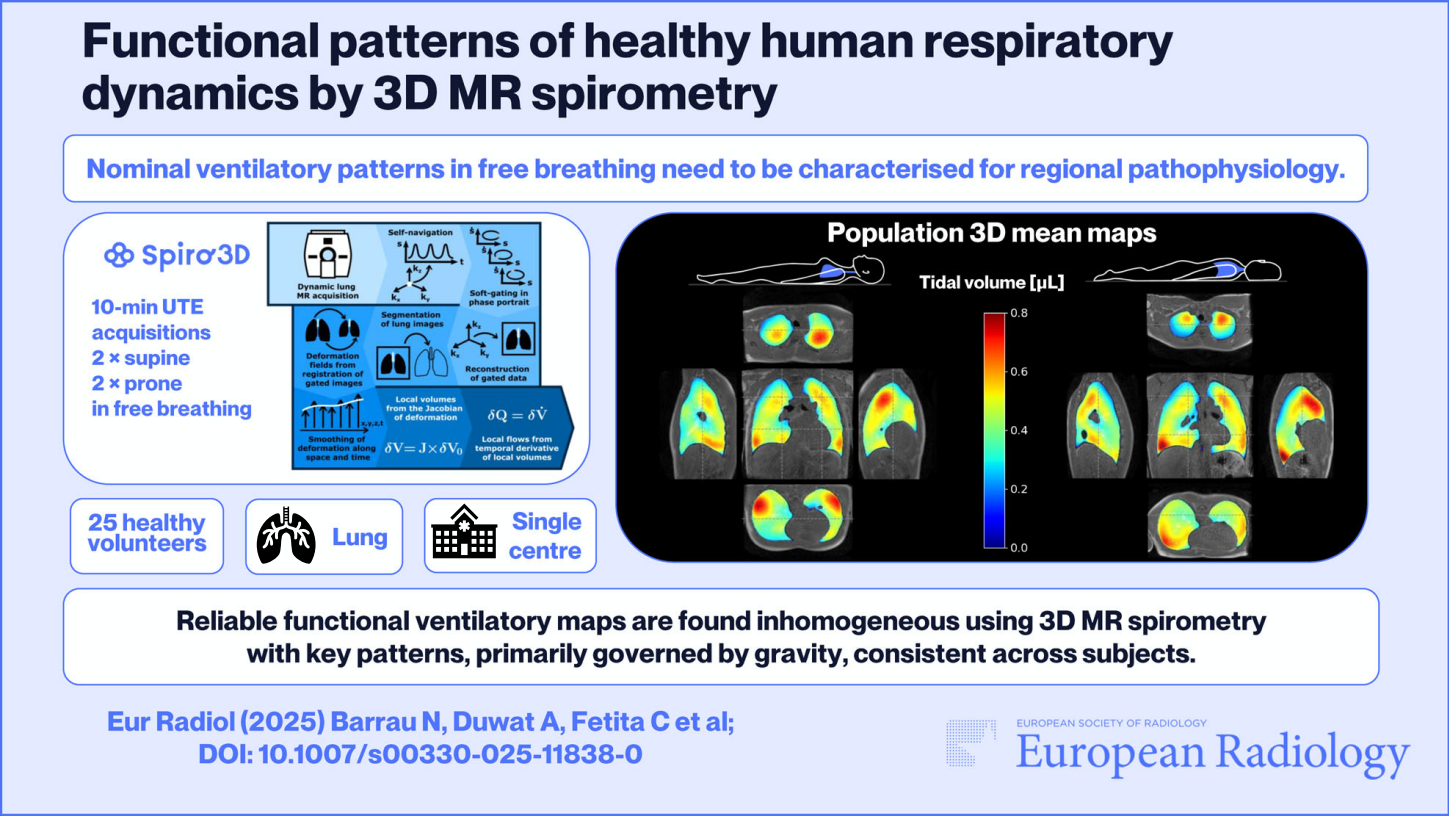

## Introduction

Standard spirometry is the reference exam to assess ventilation: from flow-volume loops at maximal breathing, clinicians can diagnose and stage obstructive or restrictive syndromes. Although spirometry remains a critical diagnostic tool, its utility is limited by the need for forced breathing manoeuvres, which are not feasible for all patients [1–3]. Additionally, spirometry provides only a global assessment of lung function. Since breathing is intrinsically a three-dimensional phenomenon [4] and lung diseases are generally regional [5], ventilation should be locally probed. Functional lung imaging advantageously complements spirometry by characterising the local ventilation.

Lung MRI offers a promising non-ionising solution for temporally resolved assessments of regional ventilation. Despite initial challenges such as low proton density, rapid signal decay, and physiological motion, recent advancements have overcome these limitations [6–8]. Using ultrashort time echo (UTE) sequences combined with retrospective gating, a series of lung images can be reconstructed over an entire respiratory cycle. Ventilation is then evaluated on these dynamic images from variations in either the MR signal intensity [9–11] or the Jacobian of the deformation field [4, 10, 11], all without the need for exogenous contrast. The former strategy relies on the strong assumption that ventilation volumes linearly evolve with the MR signal intensity. Initiating the latter, Plathow et al [10] and Kolb et al [11] made use of a shape model to infer local deformation and ventilation volumes over the respiratory cycle. Three-dimensional MR spirometry was then performed with deformation field processing based on solid anatomical landmarks [4].

The feasibility and clinical interest of functional lung MRI without contrast agents have been confirmed in free breathing [12, 13]. However, spatial patterns of the nominal ventilation were not characterised in free respiration, although required for regional pathophysiology. Moreover, since the work of Kaneko et al in 1966 [14], it has been known that gravity induces inhomogeneous ventilation with a positive gradient in the direction of gravity. Under its own weight, the parenchyma deforms under the so-called “Slinky® effect” [15], which promotes a higher alveolar density and lower pressure in the dependent region, allowing more gas to flow and diffuse along with enhanced perfusion. Here, we implement 3D MR spirometry on healthy volunteers freely breathing in both supine and prone positions to investigate nominal patterns of the respiratory dynamic. The reliability of the technique is first demonstrated by assessing its accuracy and repeatability, as well as its sensitivity to gravity.

## Materials and methods

### Study participants and design

**Fig. 1:**
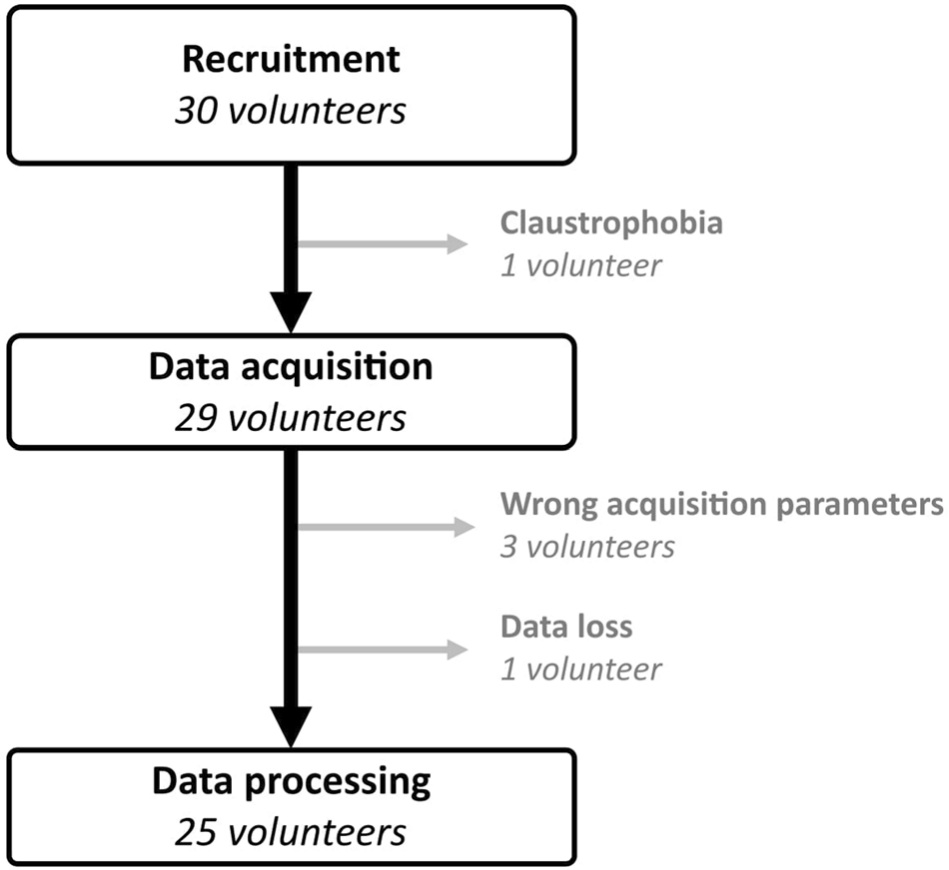
Flowchart of the study: 30 healthy volunteers were recruited, and 25 volunteers were included. All participants underwent 3D MR spirometry exams in the supine and prone positions

The prospective study was approved by the local ethics committee (CPP n°19.06.48, SI-CNRIPH 19.05.23.47941). Thirty healthy asymptomatic volunteers were recruited between February 2021 and April 2022 (Fig. 1). All volunteers received detailed information about the protocol, were included by the investigators, and gave informed consent. Inclusion criteria were the age (between 18 and 75 years old), the ability to stay inside an MR scanner without moving during the acquisition, and the absence of a known pulmonary disease. Exclusion criteria were the contraindication to MRI, and a tobacco history of more than 5 packs a year. Volunteers were asked to breathe spontaneously while they underwent four successive dynamic lung MRI acquisitions: two repeated acquisitions in the supine position, with a time interval of 5–10 min, and two repeated acquisitions in the prone position, with the same time interval. Between the supine and the prone positions, there was an interval of 20–25 min to allow for position change.

### MR acquisition protocol

The study was performed at 3 T (GE Signa PET/MR, GE HealthCare) using a UTE sequence combined with an adaptive Koosh ball adaptative zero time echo k-space trajectory (AZTEK) trajectory (speed = 31, twist = 4, shuffle = 2) [16], and a 30-channel thoracic coil array for signal reception. Acquisition parameters were set for all volunteers with TE = 14 µs, TR = 2 ms, flip angle = 3°, bandwidth = ± 100 kHz, and voxel size = 1.5 mm isotropic. The field of view was adjusted to each morphology, leading to acquisition time varying between 8 min to 12 min for a full dynamic dataset. The subject’s breathing was monitored by a pressure belt.

### Computation of 3D MR spirometry maps

**Fig. 2:**
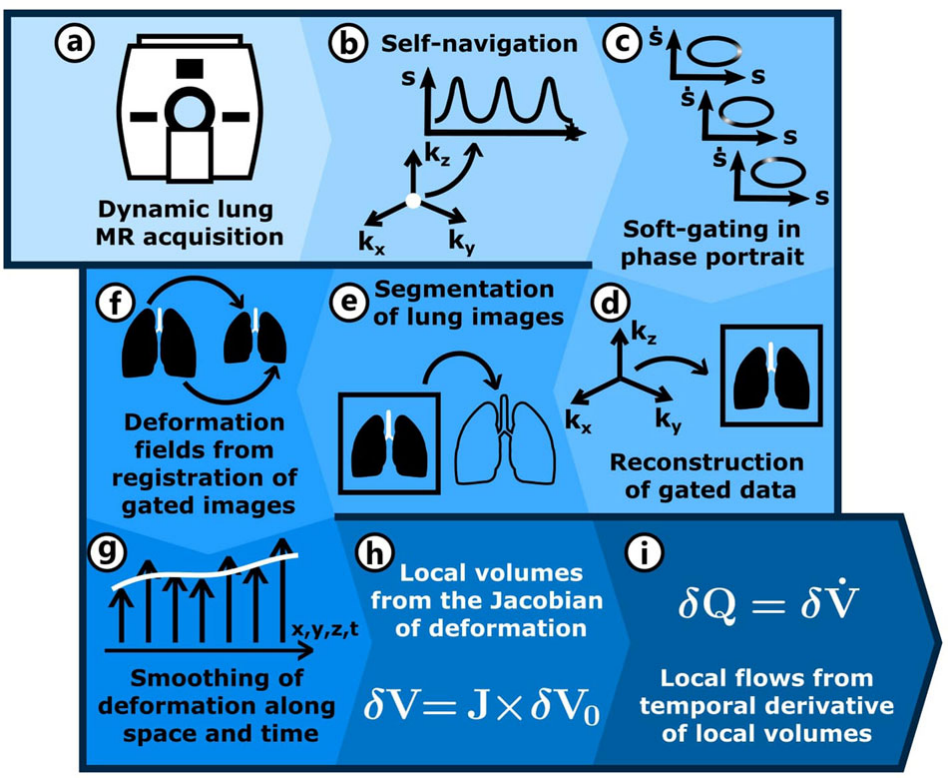
3D MR spirometry processing pipeline. After dynamic lung acquisition using an ultrashort time echo sequence (**a**), a surrogate respiratory signal is extracted from the centre of the k-space to monitor the respiration (**b**). A retrospective soft-gating is performed on the phase portrait of the respiratory surrogate to bin the MR data into 32 respiratory phases with minimal motion artefacts (**c**). Each phase is reconstructed with parallel imaging and compressed sensing (**d**). The lung volume is automatically segmented for each respiratory phase using a UNet model (**e**). Deformation fields are inferred from elastic registration (**f**) and smoothed along the temporal dimension (**g**). Local ventilatory volumes are assessed from the determinant of the Jacobian matrix of the strain tensor (**h**) and temporally derived to compute local flows (**i**). These absolute measurements of local flow-volume loops set the outputs of 3D MR spirometry

Figure 2 summarises the processing chain of 3D MR spirometry. A surrogate respiratory signal was extracted from the centre of the acquired k-space to retrospectively bin the MR data into 32 respiratory phases. Soft-gating was performed on the phase portrait of the signal to minimise motion artefacts. Each phase was reconstructed with parallel imaging and compressed sensing methods using the BART toolbox v0.4.00 [17]. The lung was automatically segmented for each respiratory phase using a UNet model fully trained on 3D UTE lung volumes within the nnUNet framework [18]. The functional residual capacity (FRC_MRI_) could be deduced from end-of-expiration after a mean lung density correction. Deformation fields were extracted from elastic registration [4] using the Elastix toolbox [19]. The elastic registration process was based on the Normalised Mutual Information metric and was performed using four different resolution steps, with the final grid resolution set to 16 isotropic voxels (24 mm). A 3D B-spline transform model was used, and the registration was optimised using an Adaptive Stochastic Gradient Descent algorithm with 100 iterations per resolution level. The deformation fields were subsequently smoothed along the temporal dimension [20, 21]. Local ventilatory volumes were inferred from the determinant of the Jacobian matrix of the strain tensor and temporally derived to compute local flows. Absolute measurements of local flow-volume loops were finally produced as 3D+t parametric maps. Ventilatory parameters, globally estimated in free-breathing standard spirometry, were evaluated here for every voxel throughout the lung [22, 23]:

- the tidal volume (TV),
- the spontaneous peak expiratory flow (SPEF),
- the spontaneous expiratory flow at 25% of the tidal volume (SEF25).

The maximum anisotropy deformation index (ADI) was also evaluated for every voxel upon the strain components of the Green-Lagrange tensor to probe the non-rigid 3D nature of the pulmonary mechanics [24].

### Reliability of 3D MR spirometry

The physiological variability of the respiratory frequency and amplitude between successive acquisitions was evaluated from the signal of the pressure belt. The measurement repeatability of 3D MR spirometry parameters was assessed globally and locally. Global accuracy of volumes was validated by calculating a global TV from segmented lung volumes (TV_seg_) and comparing it with the integrated local TVs from 3D MR spirometry. TV_seg_ was determined by comparing end-inspiratory and end-expiratory lung volumes, segmented using the UNet model. Sensitivity to gravity was assessed by comparing the anterior and posterior regions delineated by dividing the lungs into two equal volumes. Variation rates of tidal volumes between dependent and nondependent lungs were used to evaluate the extent of the gravity dependence.

### Normalisation of parametric maps

Spatial and feature scaling normalisation was applied to enable intra- and inter-volunteer comparison of 3D MR spirometry outcomes. This approach accounted for physiological variability in spontaneous breathing, as well as differences in volunteer morphology and pulmonary capacity. First, a morphological normalisation was performed using elastic registration to match the lungs of all the volunteers to the lung of a chosen reference dataset. The reference lung dataset was chosen for its detailed anatomical landmarks and distal vascular tree, which supports high-quality partition into lobes along pulmonary vessel segmentation [25, 26]. Second, each histogram was normalised to the reference dataset by globally scaling the integrated histogram values to match the integrated histogram value of the reference histogram.

### Dependencies of the regional ventilation

Despite the normalisations, additional cofactors are expected to play a role in the distribution of ventilation as standard spirometry is known to be dependent on subjects’ height, age, sex, or ethnicity [27, 28]. The influence of the lung volume [29] and the age [30] on the lung gravity dependence was evaluated. The temporal evolution between repeated acquisitions of respiratory features was also assessed for most gravity-dependent regions, as compressed alveoli or small airways can eventually collapse over time [31].

### Statistical analysis

Global accuracy of tidal volumes was quantified using the single fixed raters intraclass correlation coefficients (ICC) and their bias, evaluated from the median percentage difference (PD) [32]. Test-retest repeatability of 3D MR spirometry was assessed globally and locally, using a Bland–Altman analysis, then quantified by median PD and ICC. Inter-volunteer reproducibility of functional patterns was evaluated upon the median coefficient of variations (CV). A paired Wilcoxon test was performed to ensure the significance of the measured gravity dependence. The effects of age and lung volume on gravity dependence were assessed using Kendall’s rank correlation coefficient and Mann–Whitney test. Relationships between parameters were assessed using Spearman’s correlation coefficient. Results were considered significant when the two-tailed type I error probability (α) was less than 5% (*p* < 0.05). Statistical analysis was performed using the SciPy library (Python version 3.8).

## Results

### Population

Among the thirty healthy asymptomatic volunteers recruited for the study, one volunteer did not complete the MR exam due to claustrophobia, three datasets were acquired with the wrong imaging parameters, and one set was lost during data export. Finally, twenty-five volunteers, 10 females and 15 males, were included (Fig. 1). As reported in Table 1, the main physiological characteristics were broadly distributed with ages ranging from 18 to 75 years and body mass indices from 20 to 31 kg/m².

**Table 1:** Descriptive statistics of the population

Description of the population	Healthy volunteers (*n* = 25)
Age (years)	
Mean ± standard deviation	44 ± 17
Median ± interquartile range	43 ± 29
Range	20–75
Sex	
Female	10
Male	15
Weight (kg)	
Mean ± standard deviation	70 ± 11
Median ± interquartile range	72 ± 13
Range	54–102
Height (cm)	
Mean ± standard deviation	171 ± 9
Median ± interquartile range	171 ± 16
Range	156–185

### Global lung function measurements

Statistics on global values—namely integrated over the voxels of the lung volume—over the population and positions are provided in Table 2. Global TV of 3D MR spirometry measurements (TV) were found consistent with global TV inferred from segmented lung volumes (TV_seg_), with a median PD of 9.6% and ICC of 0.97. Mean TV of 397 mL and 455 mL are found in the supine and prone positions, while mean TV_seg_ are respectively of 433 mL and 490 mL.

**Table 2:** Global functional residual capacity (FRC_MRI_), tidal volume (TV), spontaneous peak expiratory flow (SPEF), spontaneous expiratory flow at 25% of tidal volume (SEF25), and global anisotropy deformation index (ADI) computed over 101 3D MR spirometry datasets acquired in 25 healthy volunteers with repeated acquisitions in the supine and prone positions

Global lung function	Supine	Prone
FRC_MRI_ (L)		
Mean ± standard deviation	1.85 ± 0.61	2.02 ± 0.64
Median ± interquartile range	1.78 ± 0.61	2.01 ± 0.60
Range	[0.77, 3.29]	[1.11, 3.68]
TV (mL)		
Mean ± standard deviation	397 ± 106	455 ± 145
Median ± interquartile range	370 ± 132	433 ± 197
Range	[271, 760]	[243, 838]
SPEF (mL/s)		
Mean ± standard deviation	418 ± 148	414 ± 167
Median ± interquartile range	381 ± 169	375 ± 166
Range	[229, 849]	[204, 993]
SEF25 (mL/s)		
Mean ± standard deviation	341 ± 67	339 ± 92
Median ± interquartile range	331 ± 92	325 ± 74
Range	[227, 493]	[206, 677]
ADI (× 10^4^)		
Mean ± standard deviation	9.63 ± 3.44	10.5 ± 3.5
Median ± interquartile range	9.09 ± 2.99	10.1 ± 5.2
Range	[5.6, 22.7]	[4.7, 19.3]

### Sensitivity to gravity dependence

**Fig. 3:**
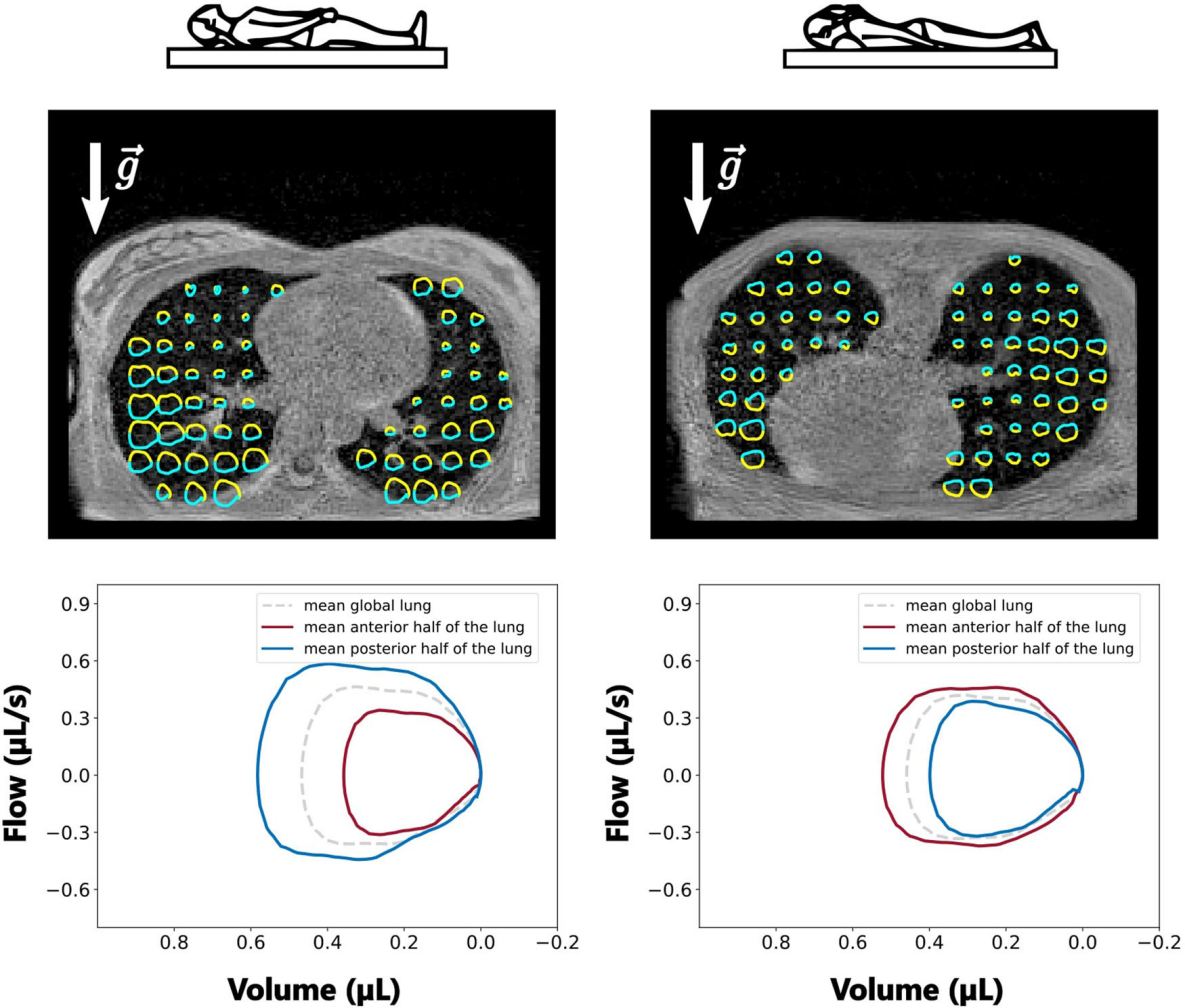
Top row: Axial maps of local flow-volume loops (one voxel out of 11 is represented) with inspiration (yellow) and expiration (cyan) phases. Bottom row: Absolute flow-volume loops on a healthy volunteer in the supine (left) and prone (right) positions. The global flow-volume loops over the lung are similar whether in the supine or prone positions, but when integrated over the anterior and posterior halves, the flow-volume loops largely differ between the dependent and nondependent regions, which reveals the sensitivity to gravity of 3D MR spirometry

The lung gravity dependence leads to higher volumes and flows in the more gravity-dependent region: the posterior region for supine and the anterior region for prone (Fig. 3). The gravity dependence is statistically significant for both positions when comparing the anterior and posterior half lungs over the 25 volunteers for TV, SPEF and SEF25 (*p* < 0.05 using a paired Wilcoxon test, Table 3). A significant difference is also found for ADI; however, the posterior region always shows higher values, whether in supine or prone positions. Parametric maps for flows and volumes are found less gravity-dependent in the prone position: in supine, TV is 37% higher in the posterior region than in the anterior region, whereas, in prone, it is 15% higher in the anterior region than in the posterior region. The right lung is found more dependent on gravity effects in the supine position (42% vs 33%), while the left lung is found more dependent in prone (24% vs 7%). Higher volumes are found in the prone position, with global TVs of (455 ± 145) mL compared to (397 ± 106) mL in the supine position (Table 2).

**Table 3:** Assessment of differences between gravity-dependent and gravity-nondependent lung for the supine and prone positions

Parameter	Position	Comparison	Absolute differences (median ± interquartile range)	Variation rates (median ± interquartile range)	*p*-value
TV					
	Supine	Posterior–anterior	(0.25 ± 0.23) µL	(37 ± 29) %	< 0.05
	Prone	Anterior–posterior	(0.12 ± 0.12) µL	(15 ± 18) %	< 0.05
SPEF					
	Supine	Posterior–anterior	(0.19 ± 0.29) µL/s	(30 ± 38) %	< 0.05
	Prone	Anterior–posterior	(0.08 ± 0.12) µL/s	(12 ± 19) %	< 0.05
SEF25					
	Supine	Posterior–anterior	(0.28 ± 0.20) µL/s	(51 ± 34) %	< 0.05
	Prone	Anterior–posterior	(0.09 ± 0.10) µL/s	(17 ± 18) %	< 0.05
ADI					
	Supine	Posterior–anterior	0.03 ± 0.03	(23 ± 14) %	< 0.05
	Prone	Anterior–posterior	−0.04 ± 0.04	(−15 ± 14) %	< 0.05

Median tidal volume (TV), median spontaneous peak expiratory flow (SPEF), median expiratory flow at 25% of tidal volume (SEF25) and median anisotropy deformation index (ADI) are calculated and compared for each volunteer’s lung halves (voxel size is 1.5 mm isotropic). Cohort statistics are presented as median and interquartile range. Statistical significance was assessed using a paired Wilcoxon test

### Repeatability of 3D MR spirometry measurements

**Fig. 4:**
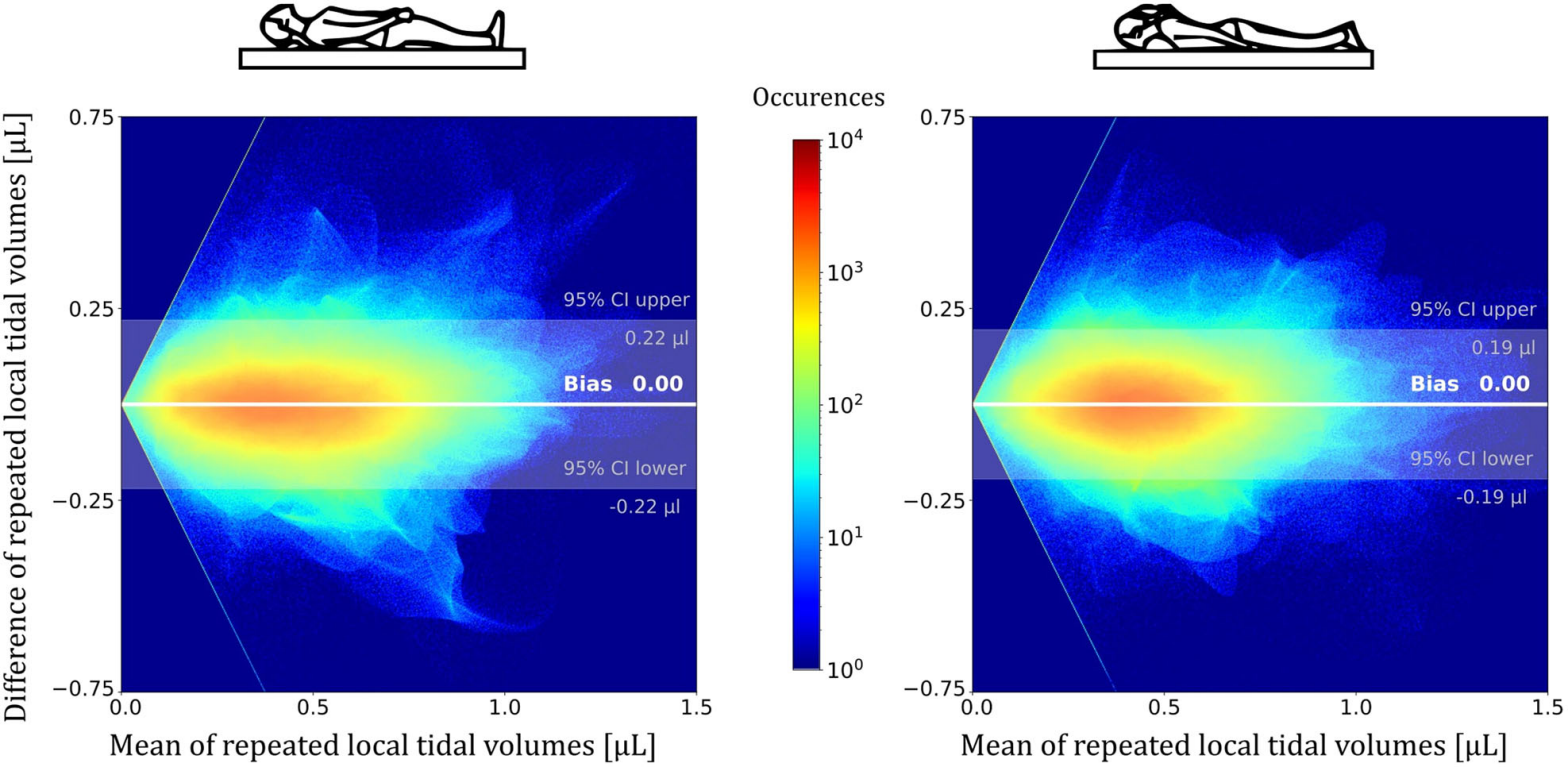
Bland–Altman analysis for normalised local tidal volumes over the 25 healthy volunteers in the supine (left) and prone (right) positions: the means of repeated acquisitions are plotted against their difference. The 95% confidence intervals (CI) are similar between positions (bleached area). There is essentially no bias

From the signal of the respiratory belt, we recorded an intrinsic variability of spontaneous breathing with median PD between successive acquisitions of 4.3% on its mean amplitude and 9.3% on its mean frequency. The same range of variability is measured on global 3D MR spirometry parameters, with median PD from 5.7 to 9.2%, and ICC from 0.71 to 0.88 (Table 4). Locally, enhanced repeatability across all parameters is found after normalisation, with median PD decreasing from [14%, 22%] to [11%, 19%], and ICC values improving from [0.71, 0.87] to [0.78, 0.90] (Table 4). The 95% confidence interval of TVs is [−0.22 µL, +0.22 µL] with a median TV of 0.43 µL and [−0.19 µL, +0.19 µL] with a median TV of 0.44 µL, for the supine and prone positions respectively (Fig. 4). For both positions combined, the 95% confidence interval of TVs is [−0.21 µL, +0.21 µL] with a median TV of 0.43 µL. Additionally, within a 15 mm thick peripheral layer and despite normalisation, local TVs are found significantly reduced in the second repeated acquisition in the most gravity-dependent region: median reductions of 6.4% in supine and 5.6% in prone (*p* < 0.05 using a paired Wilcoxon test, Table S1).

**Table 4:** The repeatability of global and local 3D MR spirometry parameters is assessed using the median percentage differences and intraclass correlation coefficients

3D MR spirometry repeatability	Global	Local	
		Before normalisation	After normalisation
TV			
Median percentage difference (%)	5.67	14.7	12
Intraclass correlation coefficient	0.77	0.82	0.87
SPEF			
Median percentage difference (%)	9.17	21.8	18
Intraclass correlation coefficient	0.71	0.71	0.78
SEF25			
Median percentage difference (%)	6.49	20.2	19
Intraclass correlation coefficient	0.88	0.81	0.81
ADI			
Median percentage difference (%)	6.38	14.0	11
Intraclass correlation coefficient	0.83	0.87	0.90

Global parameters are computed as the sum of local parameters over the lung volume. Local biomarkers are computed within each voxel of size (1.5 × 1.5 × 1.5) mm^3^. Spatial registration on a reference dataset and global feature scaling were performed to normalise the parametric maps, enabling local comparison with enhanced repeatability. This repeatability, assessed in free breathing, includes the intrinsic physiological variability

### Inter-volunteer reproducibility of functional patterns

**Fig. 5:**
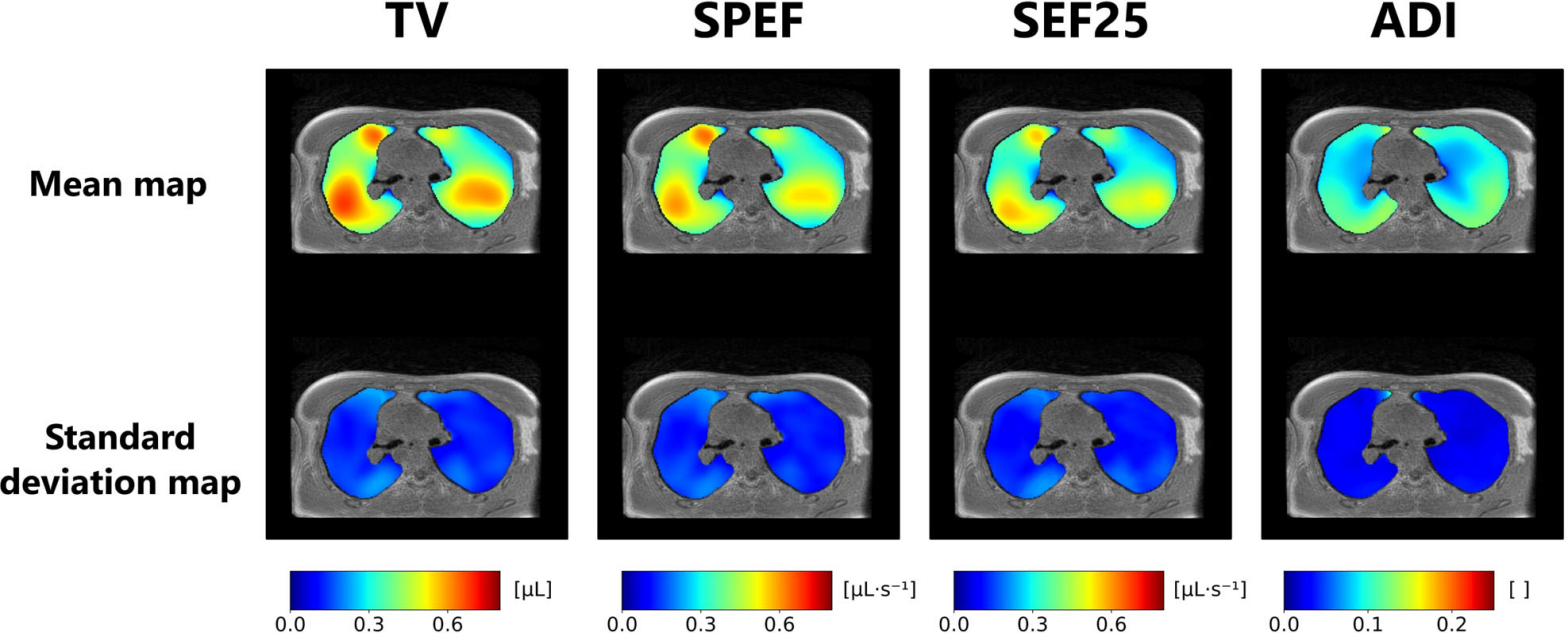
Central axial slices of mean (top row) and standard deviation (bottom row) maps of (from left to right) tidal volume (TV), spontaneous peak expiratory flow (SPEF), spontaneous expiratory flow at 25% of tidal volume (SEF25), and anisotropy deformation index (ADI), over the 25 healthy volunteers imaged in the supine position

Individual parametric maps are highly inhomogeneous over the lung volume, with large coefficients of variation ranging between 30% and 63%. Mean normalised maps for 25 healthy volunteers remain also inhomogeneous, with CVs between 25% and 32%, highlighting consistent ventilation patterns during free breathing in supine and prone positions (Fig. 5). Their good reproducibility is supported by low local variation in highly ventilated lung areas (median CVs in the top quartile of values between 11% and 13%), while overall lung CVs range from 28% to 38% in both positions. FRC_MRI_ slightly correlates with the difference of ventilation between the gravity-dependent and nondependent lungs at *τ* = 0.19 with Kendall’s rank correlation coefficient. This correlation is significant when the cohort is divided into two groups of lower and higher FRC_MRI_ (boundaries: [0.77 L, 1.90 L] and [1.90 L, 3.68 L], *p* < 0.05 using an unpaired Mann–Whitney test). No correlation is observed between gravity lung dependence and age ($\tau$ = −0.06, *p* = 0.70; groups: [20–43 years] and [43–75 years]).

### Spatial distribution of 3D MR spirometry parameters

**Fig. 6:**
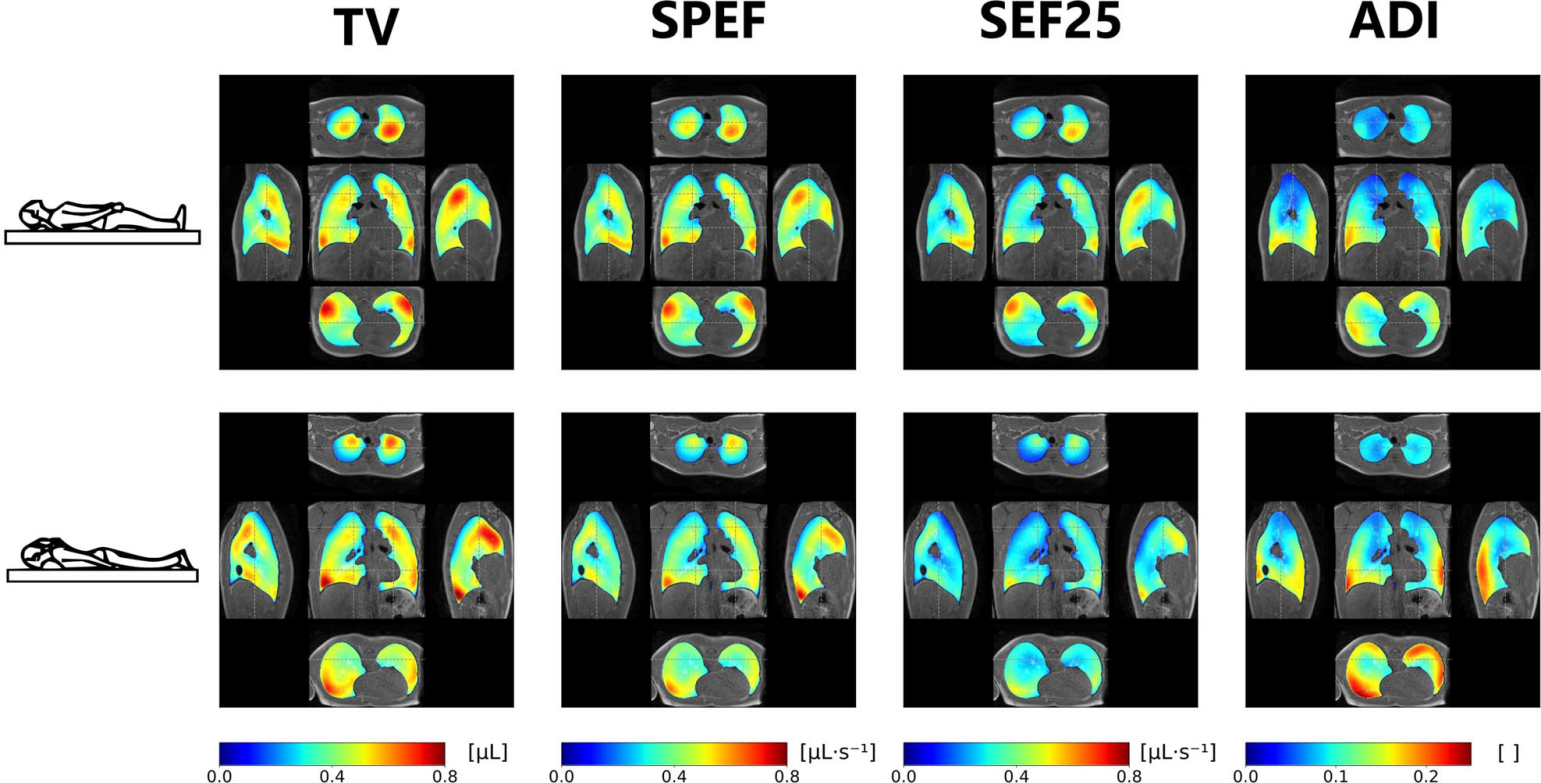
Exploded view of the parametric lung volume with a central coronal view, side right and left sagittal views, and apical and basal axial views with the same orientation in the supine (top row) and prone (bottom row) positions. Black dash lines indicate the positions of the different views. Parametric maps of tidal volume (TV), spontaneous peak expiratory flow (SPEF), spontaneous expiratory flow at 25% of tidal volume (SEF25), and anisotropy deformation index (ADI) are averaged over the 101 datasets of the 25 healthy volunteers after normalisation

Lobar 3D MR spirometry parameters in the supine position are summarised in Table S1. Before feature scaling, significant functional differences are observed across lobes (the mean lobar TV ranges from 25 mL in the right middle lobe to 125 mL in the right inferior lobe) and volunteers (ADI ranges from 1.7 × 10⁴ to 9.7 × 10⁴ in the right inferior lobe). Figure 6 highlights nominal ventilatory patterns defined from the normalisation of individual parametric maps. Higher TV values are found in the apical and basal posterior segments in the supine position, while they are in the anterior segments in the prone position. SPEF and SEF25 maps closely correlate with the TV map (r > 0.9 for both positions). The deformation anisotropy maps present different patterns, especially in the supine position, and exhibit a smaller correlation with the TV map, (r = 0.32 and r = 0.50 for the supine and prone positions respectively), with higher ADI values in the inferior and posterior segments, close to the rib cage and the diaphragm for both positions.

## Discussion

From a deformation-based analysis of a dynamic series of 3D lung images, 3D MR spirometry enables a robust assessment of local ventilation throughout the lung. Studied parameters (TV, SPEF, SEF25, and ADI) were normalised over 25 healthy volunteers to establish nominal free-breathing respiratory characteristics.

Global TV values inferred from 3D MR spirometry are validated against segmented lung volumes. Mean FRC_MRI_ values in supine and prone positions (1.9 L and 2.0 L) were shown to be close to the values measured by helium dilution (1.9 L and 2.1 L), so they could serve as an alternative measurement [33]. To our knowledge, lobar measurements of the free ventilation are presented here for the first time. The repeatability is globally limited to median percentage differences (5.7%–9.2%) in the same range as those recorded by the respiratory belt (4.3%–9.3%), which is consistent with reported global ventilation variability in healthy volunteers [34–36]. The overall repeatability of 3D MR spirometry is very good, as the measurement variation is dominated by the intrinsic variability of human free breathing. The normalisation strategy accounts, at least partially, for the variability of the volunteer’s respiration and yields good local repeatability (ICC between 0.78 and 0.90). Yet, some low values of local volumes or flows still fall within the standard deviation over the studied population, setting an upper bound for 3D MR spirometry precision. This limit reflects both the precision of the measurement and the inherent variability among individuals, all of whom are expected to have their own respiratory personalities [37].

Three-dimensional MR spirometry is sensitive to the lung gravity dependence, with a stronger effect in the supine position as previously reported in the literature [14, 38]. Moreover, repeated measurements reveal a slight decrease in ventilation over time in the more gravity-dependent region, particularly in the supine position. Greater gravity dependence may lead to a local reduction of the lumen in some airways over time, while body structure and fluid dynamics are rearranged with the change in gravity field from the standing to the supine position. In the studied 25 healthy volunteers (Table 5), the functional residual capacity appears to be a significant cofactor of lung gravity dependence.

**Table 5:** Lobar measurements of 3D MR spirometry computed over datasets from 25 healthy volunteers lying in the supine position

Lobar lung function	Left lung Superior lobe	Left lung Inferior lobe	Right lung Superior lobe	Right lung Middle lobe	Right lung Inferior lobe
TV (mL)					
Mean ± standard deviation	88 ± 26	95 ± 28	61 ± 17	25 ± 8.3	125 ± 41
Median ± interquartile range	82 ± 40	91 ± 32	59 ± 22	24 ± 9	115 ± 43
Range	[57, 160]	[55, 176]	[35, 103]	[16, 50]	[61, 269]
SPEF (mL/s)					
Mean ± standard deviation	93 ± 36	99 ± 35	66 ± 24	29 ± 10	138 ± 54
Median ± interquartile range	89 ± 43	92 ± 39	62 ± 35	27 ± 11	117 ± 57
Range	[44, 198]	[59, 211]	[34, 141]	[15, 57]	[60, 290]
SEF25 (mL/s)					
Mean ± standard deviation	71 ± 16	89 ± 21	49 ± 12	20 ± 5.0	111 ± 28
Median ± interquartile range	70 ± 28	87 ± 36	47 ± 16	20 ± 7.4	109 ± 35
Range	[50, 105]	[48, 136]	[27, 79]	[13, 32]	[64, 188]
ADI (× 10^4^)					
Mean ± standard deviation	1.7 ± 0.52	2.3 ± 0.75	1.2 ± 0.33	1.0 ± 0.50	3.3 ± 1.6
Median ± interquartile range	1.6 ± 0.68	2.2 ± 1.3	1.1 ± 0.35	0.93 ± 0.42	2.8 ± 1.4
Range	[0.95, 3.2]	[1.3, 4.5]	[0.82, 2.4]	[0.26, 2.8]	[1.7, 9.7]

Parameters are integrated over the total lung lobe volume at the end of expiration: tidal volume (TV), spontaneous peak expiratory flow (SPEF), spontaneous expiratory flow at 25% of tidal volume (SEF25), global anisotropy deformation index (ADI)

Ventilation appears highly inhomogeneous. The different contributions of lung segments during free breathing in a lying position are clearly highlighted and delineated by 3D MR spirometry. The studied functional biomarkers (TV, SPEF, SEF25) are sensitive mainly to the position in the lung with respect to the gravity direction and the proximity to the chest wall and to the main respiratory muscles. In healthy volunteers, the right lung upper lobe promotes ventilation in the apical segment in prone and the posterior segment in supine. In the lower lobe, ventilation is limited to the posterior basal segment in prone (Fig. 6). ADI does not appear to be sensitive to the gravity direction but seems primarily governed by the lung morphology and the respiratory muscles. The higher ADI values found in the basal regions result from the diaphragmatic effort that is essentially unidirectional along the superior-inferior direction (Fig. 6). So far, in ongoing studies [13], ventilation defect maps produced by functional lung MRI do not account for the nominal spatial distribution of ventilation even though it is clearly shown here that spatial inhomogeneity is inherent to free breathing [39]. Besides, the subject’s position could be imposed to preferably reveal ventilation impairments in the nominally solicited segments.

The revealed features are highly dependent on the subject’s lung morphology [29]. The integration of cofactors could be investigated to improve data normalisation while preserving ventilatory features, similarly to standard spirometry [27, 28], leading towards a generic pulmonary system. This could serve as a reference atlas so regional studies could be performed in the lobes, segments, or even voxels. Each of these regions contributes differently to the overall physiological function required for free breathing. They set the ground for a solid diagnosis of local lung impairment.

The primary limitation of this study is the limited size of the cohort, which restricts the statistical power to reveal cofactors of ventilatory patterns. The slight bias resulting in the underestimation of global TVs compared to segmented lung volumes could be due to regularisation steps (registration and temporal smoothing). Additionally, 3D MR spirometry measurements could have been compared with simultaneous measurements from an MR-compatible spirometer at the mouth, though this approach is limited to global comparison of volumes and flows, and such spirometers are not commercially available.

Three-dimensional MR spirometry is a new approach to quantitative regional lung pathophysiology in an unconstrained way while the subject is breathing freely. Despite the intrinsic variability of free respiration, reliable voxel-wise flow-volume loops are generated from a standard 10 min MRI acquisition. The associated functional maps measured in healthy volunteers highlight the inhomogeneous spatial distribution of the respiratory function during free breathing. The nominal patterns, mainly driven by gravity, respiratory muscles, and the thoracic cage, should be considered for the analysis of ventilation disorders.

## Supplementary information

ELECTRONIC SUPPLEMENTARY MATERIAL

## Abbreviations

ADI: Anisotropy deformation index
CV: Coefficient of variation
FRC: Functional residual capacity
ICC: Intraclass correlation coefficient
MR: Magnetic resonance
MRI: Magnetic resonance imaging
PD: Percentage difference
SEF25: Spontaneous expiratory flow at 25% remaining tidal volume
SPEF: Spontaneous peak expiratory flow
TV: Tidal volume
UTE: Ultrashort echo time
