# Supplementary material for: Functional patterns of healthy human respiratory dynamics by 3D MR spirometry
Source: Eur Radiol. 2025 Aug 8;36(2):1184–95. doi: 10.1007/s00330-025-11838-0 (PMC12953252; doi:10.1007/s00330-025-11838-0)
Supplement: Supplementary file 1 — ELECTRONIC SUPPLEMENTARY MATERIAL [file 330_2025_11838_MOESM1_ESM.pdf]

Functional patterns of healthy human respiratory dynamics by 3D MR spirometry

ELECTRONIC SUPPLEMENTARY MATERIAL

Table S1. Assessment of temporal variations in ventilation in a 15 mm layer of the dependent lung. Median local tidal volumes (TV) are evaluated over repeated acquisitions in the region (1.5 mm isotropic voxel). Cohort statistics are provided as median and interquartile range. Statistical significance was assessed using the paired Wilcoxon test.

**Paramete r**	**Position**	**Comparison** within 15 mm tick dependent lung	**Absolute differences** (median ± inter-quartile range)	**Variation rates** (median ± inter-quartile range)	**p-value**
**TV**					
	Supine	acq 1 – acq 2	(0.02 ± 0.06) µL	(-6.4 ± 15) %	< 0.05
	Prone	acq 1 – acq 2	(0.02 ± 0.06) µL	(-5.6 ± 13) µL	< 0.05
